# European-wide policymaking at the urban level: a qualitative study

**DOI:** 10.1093/eurpub/ckab016

**Published:** 2021-03-16

**Authors:** Julia Mueller, Lesley Patterson, Matyas Jakab, James Higgerson, Stephanie Steels, Arpana Verma

**Affiliations:** 1 Division of Population Health, Health Services Research and Primary Care, University of Manchester, Manchester, UK; 2 MRC Epidemiology Unit, University of Cambridge, Cambridge, UK; 3 Department of Social Care and Social Work, Manchester Metropolitan University, Manchester, UK

## Abstract

**Background:**

Inter-urban area (UA) health inequalities can be as dramatic as those between high and low-income countries. Policies need to focus on the determinants of health specific to UAs to effect change. This study therefore aimed to determine the degree to which policymakers from different countries could make autonomous health and wellbeing policy decisions for their urban jurisdiction area.

**Methods:**

We conducted a cross-sectional, qualitative interview study with policymakers recruited from eight European countries (*N* = 37).

**Results:**

The reported autonomy among policymakers varied considerably between countries, from little or no autonomy and strict adherence to national directives (e.g. Slovak Republic) to a high degree of autonomy and ability to interpret national guidelines to local context (e.g. Norway). The main perceived barriers to implementation of local policies were political, and the importance of regular and effective communication with stakeholders, especially politicians, was emphasized. Having qualified health professionals in positions of influence within the UA was cited as a strong driver of the public health (PH) agenda at the UA level.

**Conclusion:**

Local-level policy development and implementation depends strongly on the degree of autonomy and independence of policymakers, which in turn depends on the organization, structure and financial budget allocation of PH services. While high levels of centralization in small, relatively homogenous countries may enhance efficient use of resources, larger, more diverse countries may benefit from devolution to smaller geographical regions.

## Introduction

Globally, ∼55% of the world’s population now live in cities.^1^ Migration into cities is constantly increasing, with the world’s urban population rising from 746 million in 1950 to 3.9 billion by 2014.^1^

UAs often differ in health outcomes from the national level,^2^^,^^3^ and inter-UA health inequalities can be as dramatic as those between high and low-income countries.^4^ A key mechanism for bringing about change in health outcomes for UAs is public health (PH) policy.^5^ This includes laws, regulations, judicial degrees, guidelines and budget priorities,^5^^,^^6^ to target, e.g. alcohol control.^7^

Policies need to focus on the determinants of health specific to UAs in order to effect change. For example, spatial analysis techniques have shown that different factors determine childhood obesity depending on the socioeconomic status of the area.^8^ Developing and implementing policy at urban level can be challenging due to the diversity and complexity of UAs.^9^ Zones such as city centres, industrial, commercial and suburban areas, can differ markedly.^2^

Policies are usually developed and implemented at the national level.^10^ It is unclear to what extent UA policymakers are able to influence health policy implementation and how decisions are made at UA level. This is also likely to vary considerably between different countries, as heterogeneity within a country (e.g. in terms of socioeconomic variables, culture, languages and ethnicity) can affect levels of centralization.^11^ Understanding these complex decision-making processes is crucial to the successful development and implementation of health policy; a failure to understand and address them can lead to ineffective policies.^5^

This important question was explored within the European Urban Health Indicator System projects (EURO-URHIS 1 and 2). EURO-URHIS 1 focused on establishing a network of urban areas across Europe and developing an urban health information and knowledge system.^12^ EURO-URHIS 2 was dedicated to developing tools to help policymakers assess and improve the health of urban populations and resulted in the largest set of urban health indicators world-wide.

Results from EURO-URHIS 1 suggested that even when sub-national data are available they are often unused for local policymaking, with decisions still being made at national level.^10^ This highlights the need to understand how policy at UA level is developed and implemented as well as the political environment and incentives facing policymakers.^13^

In this study, we aimed to determine the degree to which policymakers could make autonomous health and wellbeing policy decisions at their urban jurisdiction area, across a wide variation of urban contexts in Europe.

## Methods

### Data collection and participants

We conducted a cross-sectional, qualitative interview study with policymakers recruited from eight European countries. A pilot of the interview process for this proposed study was conducted previously, in response to perceived need for further research in this area. The interview schedule used in the present study used the same questions and some that had evolved through open enquiry with participant policymakers.

In UK, Directors of PH were invited to participate. A pragmatic sampling method for recruiting interviewees from other countries was employed: EURO-URHIS 2 partners were contacted and asked to identify and recommend a senior and appropriate policymaker responsible for PH policymaking in their urban area. A researcher then contacted the potential participants directly by email or telephone. They were invited to include colleagues in the interview if they wish. Where English translation was required, participants were offered the assistance of our project partner.

Each semi-structured interview was carried out by the recruiting researcher (L.P.) as well as one other member of the research team (A.V., J.H. or S.S.) according to their availability. These researchers were all experienced in qualitative research methods. All interviews were conducted at participants’ place of work. The main focus of enquiry was the geographical level at which policymakers could make decisions about PH within the context of all healthcare provision at the UA level. Interviews were recorded and transcribed verbatim. Thematic analysis^14^ was used to analyze the data. Interview transcripts were first read repeatedly to achieve data familiarization and to generate initial descriptive codes, which were then grouped into more conceptual themes. Two researchers (L.P. and M.J.) independently undertook coding to enhance rigour and reproducibility. Discrepancies were discussed until consensus was reached. Predominant themes and sub-themes were identified and supporting quotes from policymakers are provided.

### Research context

To contextualize our findings, table 1 provides an overview for each of the eight countries, including respective geographical and population sizes, and a brief summary of devolution levels and where responsibilities for PH lay at the time of interview.

**Table 1:** Contextual information on countries included in the present study

Country	European region	**Country population size** ^a^	Country geographical size (km^2^)^a^	Brief contextual information on devolution level
Slovak Republic	Central	5 426 252	29 035	The public health network is overseen by the Ministry of Health and is financed solely from state budget. The 36 regional Public Health Institutes act as executive bodies of the Public Health Authority (PHA), which is responsible for initiating public health measures and legislation.^15^
Romania	South-Eastern	19 511 000	238 391	The Ministry of Health assumes responsibility for principal public health service guidelines. District Public Health Authorities are granted responsibility for the provision of public health services locally.^17^
Lithuania	Northern	2 827 947	65 300	The Ministry of Health assumes responsibility for principal public health service guidelines. Municipal public health bureaux are responsible for various local functions, such as implementation of local public health programmes, and population health monitoring.^16^
Slovenia	Central	2 065 879	20 273	National Institute of Public Health and nine regional public health institutes primarily responsible for public health. Public health initiatives at local level often funded by alternative sources (public and private).^19^
Latvia	Northern	1 953 200	64 589	Municipalities can implement and finance local initiatives, and practical health promotion work is often commissioned to municipalities.^20^
Netherlands	Western	17 100 475	41 543	Final responsibility for the health sector lies with the government. Public health functions fall under the authority of municipalities.^38^
Norway	Western	5 258 317	385 178	Responsibility for public health rests with the Ministry of Public Health and various other central bodies, but public health activities are implemented and executed at municipal level, and municipalities are also expected to collect data regarding their population’s health, and use this to inform their public health strategies.^39^
UK	Western	54 786 300	130 279	Responsibility for public health primarily falls under the Department of Health (DoH), but the public health services are delivered via various departments, bodies and Local Authorities (LAs). There are nine regional public health groups, and 10 strategic health authorities, through which the DoH operates at a regional level.^40^

a http://www.worldatlas.com, last accessed 08 March 2021.

## Results

Twenty-three interviews (12 with policymakers from UK, 11 with policymakers from other countries) were conducted in eight countries with a total of 37 subjects. The interviews were representative of North/Central/West/South-Eastern regions in Europe.

Interviews were typically 1–1½ h in length. Interviewees mainly elected to be interviewed on their own (in 14 UAs) or with one additional senior colleague (in 5 UAs); in three instances they included several colleagues (PM18 = 6 participants; PM24 = 3; PM43 = 4).

We aimed to recruit the most senior PH representation for the UA jurisdictions and this was achieved in all but one instance. This exception was an interview with senior representatives of a regional PH Bureau. However, they were very familiar with their UA equivalent institution. The lead contact interviewees were, variously, Directors and Deputy Directors of City Council/Municipal/Regional Departments or Institutions with specific responsibility for PH or overarching responsibility in Health and/or Welfare/Social Care.

### Theme 1: autonomy—degree of ability to influence PH policymaking at UA level

For all UAs, healthcare was the overall responsibility of national government with responsibility for the delivery of some aspects devolved to local or regional levels. All but two of the UA representatives indicated that they could influence health policymaking at the UA level to some degree (table 2).

**Table 2:** Autonomy—degree of ability to influence Public Health policymaking at UA level

Country	Theme 1 (T1): autonomy—degree of ability to influence PH policymaking at UA level (sub-themes T1.1–T1.6)
Slovak Republic	T1.1: no autonomy—adheres strictly to national directives but UA health agenda planned
Romania	T1.2: very little autonomy—prohibitive structure for divergence from national directives
Lithuania	T1.3: very little autonomy—expressed little need to diverge from national directives
Slovenia	T1.4: some autonomy—compliant with all national directives for health but UA driven inter-disciplinary PH
Latvia	T1.5: high degree of autonomy—increasingly able to interpret national directives to local context
Netherlands	T1.6: long established high degree of autonomy—able to interpret national guidelines to local context
Norway	
UK	

Overall, the greater the influence of a centralized government and/or the lesser the time since devolution to local jurisdiction for PH policymaking, the lesser the reported satisfaction with, and perceived effectiveness of, the response to local PH challenges. All policymakers reported a preference for using their allocated budgets flexibly in response to local needs, but for those with a greater degree of autonomy, dissatisfaction was expressed about hold-ups due to local-level bureaucracy.

#### Theme 1.1: no UA autonomy

Key informants of UAs from one country reported being unable to influence health policymaking at UA level and adhering uncompromisingly to the national directives.

*So the city absolutely does not have any way of changing the policies of the government. […] They do not have an agenda on health it is [all] at the … state level. (Slovak Republic)*

#### Theme 1.2: very little UA autonomy: prohibitive centralization

Another country’s UAs reported little autonomy but had a mechanism whereby approval needed to be sought for some level of adjustment of the national directives at the UA level. This policymaker found this situation laborious; they expressed the need for adequate funding and release from over-restrictive, centralized accountability.

*We would very much like to be decentralised [and] would be extremely pleased [to] establish some priorities in … implementing public health policies without approval every time for everything. (Romania)*

#### Theme 1.3: very little UA autonomy but little expressed need

Although one UA had established mechanisms for making independent PH decisions at the UA level that they exercised to some degree, the policymaker described a burdensome two-step process of gaining approval to diverge from national directives and guidelines. Despite this the policymaker reported little need to diverge from the national guidelines and rarely did so in practice. However, they cited a particular problem that they would like to be able to effect at a local level.

* … that example that we have about [the proximity of] schools [to places that have] alcohol licences this is … where we could intervene if we had more freedom … maybe freedom is not the right word. More power [is what we need]. (Lithuania)*

#### Theme 1.4: some UA autonomy

Another UA’s policymaker described their institution as primarily compliant with national guidelines but indicated a significant degree of autonomy in formulating and implementing UA interventions. This policymaker’s institution had responsibility for both health and social care for their UA and they also indicated strong working relations within other municipal departments.

*… a lot of prevention is on the local level and … is carried out in clinics and other health institutions and a part of it is carried out by NGOs which are co-financed by the municipality … there are workshops that deal with prevention in terms of how we eat, how we stay active and to deal with alcoholism, diabetes … (Slovenia)*

#### Theme 1.5: high degree of UA autonomy

One UA policymaker explicitly reported experiencing ongoing and increasing transition to greater UA autonomy for both primary and PH care. They cited World Health Organisation and European Commission initiatives that provide funding and credence to their work as being significant drivers for positive change. This UA had, in the past four years, moved from strong centralized control to greater local autonomy. The interviewee reported a positive outlook on this but with fears about the lack of in-country funds to support their efforts at the local level.

*The municipality is co-financing these parts … especially in public health [and] in primary healthcare it’s depending more and more on the municipality level so it’s more and more in terms of each municipality what the budget pay (Latvia)*

#### Theme 1.6: long established high degree of UA autonomy

Western European policymakers reported a high degree of long-established responsibility for PH at the UA level. They adhered to national health policy but reported an ability to make local interpretations of directives and guidance.

*The public health area [is] mostly … organised at the local level by regulations at national level … [our local plan is] based first of all on the plan of the national health level and then we [look at] the situation in [UA] and see what kind of problems we have here adding to the directives already given by the national level … And try to identify risk groups, target groups [etc] … and then we sort of formulate an idea where we want to end up. (Netherlands)*

In UK, local authorities (LAs) were experiencing a considerable upheaval during the period of the interviews (2012) as responsibility for PH services transitioned from the National Health Service (NHS) to LAs. Policymakers from UK generally expressed an expectation that the flexibility for interpretation of national directives would continue to hold sway post transition. They expressed concern about cutbacks for both health services and LAs but hoped that transition to LAs would provide ‘economies of scale’ for PH activities via integrated working with departments connected with the wider determinants of health.

*So the biggest issue that we face is depletion of the resource base … the workforce is a big issue … we’ve lost some of the best in the transition … clearly there is the issue of diminishing resources. (UK)*

### Theme 2: political perspective acting as a barrier to implementation of local policies

This theme emerged through all of our interviewees’ responses. Elected politicians, at both local and national level, were perceived as reluctant to implement evidence-based policy decisions where the consequences might be seen to be unpopular.

*There was a … demand … initiated by … [the] Ministry of Healthcare and municipalities were given the task to decide what is the minimal distance from schools, educational institutions … to open the shops to have licence to sell alcohol and schools suggested that it should be around between 500 metres to 2 kilometres. When politicians, local politicians [discussed] that it was just reduced to 50 metres … This kind of shows where they will prioritise their decisions. Is it health or is it commerce? Business wins. (Lithuania)*

### Theme 3: importance of regular and effective communication especially with politicians

We asked our participants how best to present data to effect changes at UA level, and many responded with comments about the need for regular and effective communication, and the importance of targeting specific groups pro-actively. Effective communication was uniformly emphasized as needing to be presented in a short, accessible format, e.g. in form of real-life exemplars of people facing specific PH challenges.

*Really, the simpler, the better without it being dumbed down … but presentation simplified [highlighting] key messages [and with] strong narrative to accompany the data … and analysis … that makes it accessible. (UK)*

### Theme 4: qualified and engaged health professionals enhance PH agenda facilitation

Having qualified health professionals in positions of influence within the UA was cited as a strong driver in the ability to promote and/or sustain the PH agenda at the UA level.

*Our head of department is very energetic. She is a [an academic and vocational] doctor and so … understands the health level and politician level so she is trying to reach the politicians and go on for the [health] targets. (Latvia)*

## Discussion

This study explored the degree to which it was possible for policymakers to make autonomous health and wellbeing policy decisions for their urban jurisdiction area. We identified considerable variation in the autonomy of policymakers at the urban level. Political perspectives often acted as barriers to implementing evidence-based local policies. Facilitators included regular and effective communication with experts, local politicians and non-medical stakeholders as well as having qualified health professionals in positions of influence within the UA.

### Autonomy and PH structures

Levels of autonomy varied from no autonomy and a strict adherence to national directives, to high levels of autonomy, where policymakers had the authority and capacity to interpret and tailor national directives to the local context.

The lowest level of autonomy was reported by policymakers from Slovakia. The hierarchical structure and centralized budget allocation involved in the initiation and funding of PH measures in Slovakia^15^ (table 1) likely contributed to policymakers’ perceived low degree of autonomy. Similarly, Romania and Lithuania, where policymakers also reported low levels of autonomy, have centralized structures (table 1). The latter two countries, however, have more regional responsibility than Slovakia, with District PH Authorities (Romania) and municipal PH bureaux (Lithuania) granted responsibility for local PH programmes and services.^16^^,^^17^

In larger countries, it can be costly (in terms of administrative costs) and difficult (due to a greater diversity of preferences, culture, languages and identity) to centralize decision-making.^11^ In small countries with relatively homogenous populations centralization can be easier to implement and more efficient in terms of resources. Thus, it was unsurprising to find that relatively small countries like Slovakia and Lithuania should have lower levels of autonomy. In Romania, which covers a large geographical area with almost 20 million inhabitants and 20 different minority languages,^18^ a strongly centralized structure seems less justified.

Policymakers from Slovenia and Latvia reported considerable autonomy. In both countries, the responsibility and funding for PH is shared between national and local institutions (table 1), indicating a less centralized structure and less financial dependence on central government funds than in Solvakia.^19^^,^^20^ This may have contributed to the higher perceived degree of autonomy among Slovenian and Latvian policymakers. Since the interviews were conducted, PH institutes in Slovenia have been restructured, involving an increase in the number of regional units,^19^ indicating a further shift towards more autonomy for municipalities.

Policymakers from the Netherlands, Norway and UK reported a long established, high degree of autonomy. Given the countries’ relatively larger size in terms of population and/or area (table 1), low levels of centralization were expected in these countries.^11^ PH in UK was undergoing a considerable restructure at the time of the interviews, as responsibility was transitioning from the NHS to LAs,^21^ with consequent uncertainty regarding future levels of autonomy in interpreting national directives. This reform was evaluated in a 2015 King’s fund review and found to have had ‘damaging and distracting’ effects, due to ‘top–down reorganization’ with decisions made at a high, centralized level rather than driven by the wishes and needs of health professionals and patients.^22^

### Striving for greater autonomy

Policymakers from three countries in our study reported no or very little autonomy in implementing local policies. In the Slovak Republic, the interviewee described efforts to create and promote city-led initiatives. In the countries where very little autonomy was reported (Romania and Lithuania), interviewees described laborious and restrictive processes required to change policy implementation for the local level, suggesting a wish for greater flexibility. While one interviewee reportedly felt little need to adapt national policies, they did identify a local issue in which greater autonomy would be beneficial.

Greater autonomy is linked to an enhanced ability to effect change when local, specific problems can be targeted.^23^ It should be noted, however, that high levels of local autonomy may not always lead to improvements in PH initiatives. For example, a US-based study which examined differences in evidence-based decision-making among local health departments found considerable variations, and this was related to training and expertise within the workforce.^24^ Thus high levels of autonomy coupled with limited or no relevant training among the policymaking workforce could potentially lead to implementation of strategies that are not evidence-based. Additionally, in our study those with a greater degree of autonomy expressed dissatisfaction with hold-ups due to local-level bureaucracy.

### Barriers and facilitators to policy implementation

Policymakers commented on barriers that prevented them from implementing evidence-based policies in their urban jurisdiction areas. The main barrier was the tendency of politicians to drive forward popular, rather than evidence-based, initiatives. This is supported by the literature.^25^^,^^26^ Indeed, policymakers themselves can also be ideologically biased.^27^ In order to ensure the popular choice is also the health-promoting choice, it is necessary to mobilize the public, e.g. through streamlining of public information and strengthening of media advocacy.^28^ PH approaches need to focus not only on communication between politicians and health professionals but also include the general public in the discussion, including collaboration between diverse stakeholders from various sectors.^28^ Research also emphasises the role of the media in shaping public opinions about policies^29^ and suggests that a more independent media that takes a more critical stance towards industry perspectives is required.^30^

### How can policy implementation at UA level be improved?

Evidence alone is not sufficient to drive forward effective policies that will protect and promote PH.^31^^,^^32^ Interviewees in this study made several suggestions for improving policymaking at urban level.

Participants suggested that policymaking at UA level could be improved by regular and effective communication with local politicians and other stakeholders. Participants emphasized that communication of evidence needs to be short and accessible (key points, lay language) in order to facilitate translation into policy.^33^ Policymakers also suggested that evidence is more effectively communicated when accompanied by meaningful narratives, particularly real life examples of people facing PH challenges. Research has shown that a combination of statistical and narrative evidence is most likely to lead to attitude change,^34^ and that narratives can help to illustrate how evidence is meaningful to individual people.^35^ Moreover, research suggests that evidence is most effective when tailored to the specific constituents of respective policymakers, by expressing data in ways that are meaningful to the recipients and highlights how it is relevant at the local (voting district) level.^33^

Another common theme expressed by policymakers was that qualified health professionals in positions of influence within the UA can lead to improved policymaking. It is well established that integrating policies into routine daily healthcare practice involves major difficulties.^36^ Previous research has emphasized that policies are more likely to be implemented successfully if they take the experience and knowledge of healthcare providers into account, and when they are supported and endorsed by providers.^37^

## Conclusion

Policy development and implementation at the urban area level depends strongly on the degree of autonomy and independence of policymakers, which in turn depends on the organization, structure and financial budget allocation of PH services. Where the specific challenges and the demographic profile of populations in urban areas differ from the general population at the national level, policymakers require the ability to interpret and tailor national directives to their local areas. Our findings indicate that the degree of influence of centralized governments, and the amount of time since devolution to local jurisdiction for PH policymaking, influence policymakers’ satisfaction with, and perceived effectiveness of, the response to local PH challenges.

In order to make informed decisions regarding best policies for unique local conditions and circumstances, policymakers need local-level evidence. However, evidence alone is insufficient. To overcome barriers such as political perspectives, which often lead to popular rather than evidence-based choices, policymakers need to promote long-term engagement of diverse stakeholders, including members of the public, political leaders, the private sector and the media. Successful engagement of stakeholders, particularly politicians, will require regular and effective communication, presented alongside narratives that highlight relevance to local constituents. Having qualified health professionals in positions of influence within UAs can be an important driver for implementing PH policies and interventions at the local level. In conclusion, if we want to promote local-level policymaking, we need not only local-level data but also strategies to present the evidence in a way that highlights the relevance to both local residents and local issues.
